# Adaptive Remote Sensing Image Enhancement for KOMPSAT Imagery

**DOI:** 10.3390/s26051467

**Published:** 2026-02-26

**Authors:** Giwoong Lee, Jingi Ju, Minwoo Kim, Jeongyeol Choe, Jaeyoung Chang, Kwang-Jae Lee

**Affiliations:** 1 IOPS Co., Ltd., Daejeon 35223, Republic of Korea; jingi.ju@i-ops.co.kr (J.J.); kimminu09@i-ops.co.kr (M.K.); feychoe@gmail.com (J.C.); 2 Korea Aerospace Research Institute, Daejeon 34133, Republic of Korea; jychang@kari.re.kr (J.C.); kjlee@kari.re.kr (K.-J.L.)

**Keywords:** satellite imagery, image denoising, reinforcement learning, image segmentation

## Abstract

Remote sensing images are often degraded by atmospheric effects, low illumination, and off-nadir viewing, which reduces the segmentation performance of deep models. KOMPSAT (Korea Multi-Purpose Satellite) imagery suffers from quality degradation because the Korean Peninsula is surrounded by sea on three sides and is subject to frequent weather and atmospheric variations. In practice, operators apply heuristic image enhancement techniques by hand, but these approaches are labor-intensive and inconsistent. To address this issue, we have proposed Adaptive Remote Sensing Image Enhancement (ARSIE), an automated reinforcement learning–based framework that improves segmentation performance on degraded KOMPSAT imagery. ARSIE takes only an existing segmentation network and training data as input, and learns, for each image, a sequence of enhancement operations selected from a filter pool. The policy network uses intermediate feature maps from the segmentation model to choose the next operation, ensuring that enhancement decisions directly support downstream segmentation performance. Experimental results show that ARSIE automatically discovers image-specific enhancement combinations and consistently improves segmentation accuracy on degraded KOMPSAT imagery. We demonstrate that ARSIE has the potential to be extended to improving the quality of other satellite imagery.

## 1. Introduction

In computer vision, especially in remote sensing image analysis, adverse and degraded conditions such as low-light, fog and rain are major factors that severely degrade the performance of high-level vision recognition tasks like segmentation and detection. Since remote sensing imagery (satellite imagery) can cover vast earth areas, spatial correlations play a crucial role [1]. However, images acquired at night or under poor weather suffer from severe noise, low contrast and color distortion, which fundamentally hinder the segmentation model from identifying objects and areas. In particular, the data acquisition process in Korea is vulnerable to this problem. Because the Korean Peninsula is surrounded by seas on three sides, weather and atmospheric conditions often introduce noise in optical sensors. As a result, the quality of remote sensing imagery is frequently degraded. We show that our proposed method is practical to Korean remote sensing imagery by evaluating the quality improvement from KOMPSAT imagery.

Traditionally, a common way to mitigate this issue is to apply histogram equalization (HE) during the data pre-processing stage. HE redistributes the pixel intensities to enhance image contrast. Prior studies [2,3,4,5] report that various HE-based methods, such as CLAHE (Contrast Limited Adaptive Histogram Equalization) [6] and BBHE (Brightness Preserving Bi-Histogram Equalization) [2], can effectively improve the visual quality of remote-sensing imagery. These methods often make degraded images more interpretable to humans and can benefit downstream computer vision tasks.

More recently, remote-sensing research has also explored learning-based strategies to cope with the lack of clean references and strong appearance variations, including self-supervised blind-spot learning [7] and interactive temporal correlation modeling to suppress pseudo-variations [8]. Motivated by these trends, we focus on task-driven enhancement that directly improves semantic segmentation performance without requiring original images during training.

To achieve greater performance improvements, prior studies [4,9] employ hybrid filtering, in which two or more filters are used in combination. These studies show that such combinations enhance image quality and, in turn, benefit image segmentation. However, applying multiple filters may lead to issues such as the image becoming excessively brighter or darker. Ref. [10] shows that applying multiple filters does not always improve performance, and that a specific combination of filters was found to be suitable for the dataset.

Despite the effectiveness of hybrid filtering, existing approaches still face practical limitations when deployed on diverse remote-sensing imagery. First, the optimal filter combination is often dataset-dependent, and exhaustive search over combinations quickly becomes infeasible as the number of candidate filters increases. Second, applying multiple filters without adaptation can easily introduce over-enhancement artifacts (e.g., overly bright or dark results), which may degrade downstream segmentation performance. Moreover, many enhancement pipelines implicitly assume access to reference (original) images or rely on fixed, hand-tuned filter parameters, making them less suitable for real-world scenarios where degradation patterns vary and clean references are unavailable.

These observations motivate an adaptive enhancement framework that (i) dynamically selects an appropriate *sequence* of filter operations for each input image, (ii) controls filter strengths to avoid over-enhancement, and (iii) directly optimizes enhancement for semantic segmentation performance rather than generic image-quality metrics. To this end, we introduce an RL-based model that learns image-specific enhancement policies, bridging the gap between heuristic hybrid filtering and task-driven enhancement.

Our contributions in this paper are below:

- We have proposed a Reinforcement Learning (RL)-based adaptive image enhancement model which finds a “sequence” of filter operations to improve the performance of segmentation models.
- We also find parameters of a filter (continuous values) at the same time. We do not need an additional phase to find parameters of a filter.
- Although our experiments were conducted solely on KOMPSAT imagery (Satellite Imagery), the results indicate that the proposed model can be extended and applied to other image domains in the future.

The following Section 2, Section 3, Section 4 and Section 5 are Related Works, Methods, Experimental Results and Conclusions.

## 2. Related Works

### 2.1. Image Segmentation in Satellite Imagery

Remote sensing imagery poses unique challenges for semantic segmentation tasks due to its large spatial extent, multi-/hyper-spectral characteristics, and strong spatial autocorrelation. Deep convolutional encoder–decoder architectures and their variants have become the primary choice for high-resolution aerial and satellite scenes, demonstrating substantial gains over traditional pipelines on public benchmarks [11,12,13]. Despite these improvements, segmentation accuracy remains highly sensitive to data acquisition conditions (e.g., low-light, fog, rain), which depress signal-to-noise ratio and contrast and thereby disturb to detect fine object boundaries and small structures essential for mapping tasks [13].

Classical contrast enhancement methods—e.g., histogram equalization and its local variant CLAHE—are often used as generic pre-processing to mitigate low contrast before segmentation. CLAHE limits local contrast amplification to suppress noise over-enhancement and has been widely adopted across imaging domains [6,14]. However, fixed pre-processing is agnostic to scene-specific degradations and task objectives; it may make the image overly brighter or darker in certain regions and can even be harmful for semantic segmentation if not tuned to the dataset and weather/illumination regime. This motivates adaptive, task-aware enhancement strategies that respond to individual images with unique degradations and the needs of the downstream model.

Beyond network architectures, segmentation performance can be improved through training strategies such as Masked Supervised Learning (MaskSup), which introduces random masking during supervised training to promote contextual reasoning and robustness [15]. Our method is orthogonal to this line of work, as it improves segmentation via adaptive input enhancement without relying on clean reference images.

### 2.2. Deep Reinforcement Learning for Image Enhancement

Recent work formulates low-light enhancement as a Markov Decision Process (MDP), learning sequential policies to adjust image-specific curves or to select restoration operators. ReLLIE learns a lightweight policy that iteratively estimates pixel-wise curves under non-reference rewards, yielding customizable enhancement under diverse low-light conditions [16]. RL-Restore constructs a toolbox of restoration tools and trains an agent to select a sequence of actions (including early stopping), becoming robust to unknown or mixed degradations [17]. PixelRL further demonstrates pixel-wise agents with local interactions for denoising, local color enhancement, and restoration, highlighting interpretability of the learned operations [18]. AdaptiveISP demonstrates that the filter-selection agent helps object detection model to improve its performance, while adverse images are processed through selected filters with appropriate sequences [19]. RL-SeqISP is a similar work to the proposed method. It is a task-specific image enhancement model based on RL but it fixes the sequence of ISP operations and finds parameters of ISP [20].

While these DRL paradigms are promising, they are largely developed for natural photography and optimize perceptual/quality metrics rather than downstream recognition. For remote sensing image segmentation under adverse and degraded conditions, a task-aware agent that (*i*) selects both the type and parameters of enhancement operators (e.g., CLAHE, BBHE and denoising filters) and (*ii*) is trained with segmentation-aware rewards (e.g., the change in mIoU) offers a principled path to bridge low-level enhancement and high-level segmentation performance. Our work follows this direction by learning an adaptive filter-selection policy tailored to the dataset’s degradation patterns and the segmentation model’s requirements.

## 3. Methods

ARSIE, the proposed method consists of policy network to select a sequence of actions (filters) and the segmentation network. To fully utilize the segmentation network, we extract features from the backbone network of the segmentation model. And our policy trains suitable sequences of actions by adapting the changes in features from filtered images. First of all, we formally introduce the notations. There are RL related notations: we write state, action, reward, action-history, transition as $S_{t}$, $a_{t}$, $r_{t}$, $h_{t}$, $T(S_{t+1}|S_{t},a_{t},h_{t})$ at processing timestamp *t* respectively. We have *N* filters and the set of filters $F:=\{f_{1},f_{2},...,f_{N}\}$ which contains *terminate*, a stopping operation. $N_{avail}$ means the maximum number of available actions. We intend our RL agent to use not all filters but some appropriate filters. I is an input image and $I_{e}$ is a final processed image. Following two subsections explain the network architecture of the ARSIE policy and RL environment setup including how to design reward of the environment.

### 3.1. Policy Network Architecture

As above mentioned, we utilize the backbone network of the segmentation model to extract features maps. From now on, we write the segmentation model and the backbone of it as ϕ and $\phi_{bb}$. Normally speaking, the feature maps of $\phi_{bb}$ are treated as semantic essences of an image. However, recent backbone networks yield multiple stages of feature maps; early stage features have contexture information such as edges and last stage features have semantical meaning with low-resolution. Therefore, we carefully choose the feature maps which are helpful to detect the changes in images before and after filtering and also capture semantical meaning simultaneously. For this reason, we use feature maps from the first two stages and the final stage of $\phi_{bb}$—ResNet’s $C_{2}$, $C_{3}$, and $C_{5}$ [21]. We extract them in the fixed order $C_{2}$→$C_{3}$→$C_{5}$. Hereafter, we follow the standard ResNet notation and refer to the early-stage feature maps as $C_{2}$ and $C_{3}$, and the final-stage feature map as $C_{5}$.

**Figure 1:**
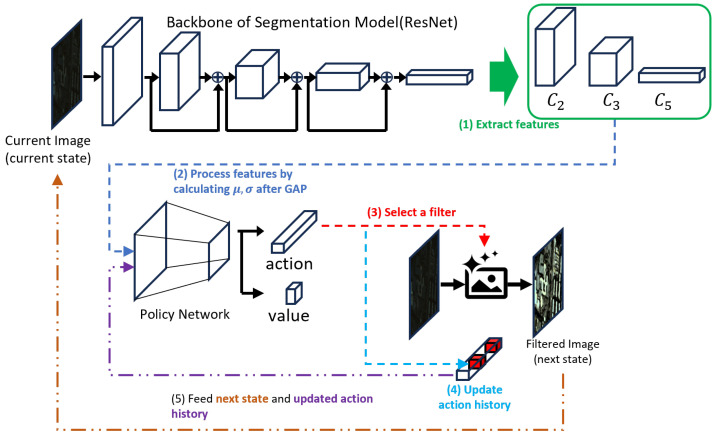
This figure shows the overall architecture of ARSIE consisting of an RL-based learning method with the backbone network. This figure is based on the description and examples in the Section 3. (**1**) shows extracting the feature maps of $C_{2},C_{3}$ and $C_{5}$ from the backbone network. (**2**) describes that GAP-pooled features and features summarized via channel-wise standard deviation serve as inputs to policy network. (**3**) means that the selected filter from action output vector produces the filtered image from the current input image. (**4**) Action-history vector is updated by recording information of previously selected filters. (**5**) The filtered image becomes next-state of RL agent and will be applied by one of the other actions (filters) including termination. Action-history and the filtered image serve as input to the ARSIE agent.

The policy network (π) uses these feature maps as inputs of the network. Firstly, we get Global Average Pooling (GAP) [22] of $C_{2},C_{3}$ and $C_{5}$; $\mu_{2},\mu_{3}$ and $\mu_{5}$; and standard deviation of $C_{5}$, $\sigma_{5}$. Global average pooling (GAP) yields compact descriptors from multi-level features: $C_{2}$/$C_{3}$ primarily encode local edges and mid-level context, whereas $C_{5}$ captures global semantics. As $C_{2}$ and $C_{3}$ are sensitive to both degradations and noise, we overcome this limitation by utilizing GAP and standard deviation of $C_{5}$ [23,24,25]. $\mu_{5}$ is relatively insensitive to additive noise, while $\sigma_{5}$ is informative about degradations, as degraded images often exhibit background equalization and contrast reduction that suppress feature variance. After extracting these features from the backbone network, we concatenate $\mu_{2},\mu_{3}$ and $\mu_{5}$ and $\sigma_{5}$. After concatenation, we apply Layer Normalization (LN) [26], and feed the normalized feature as the first input to the policy network. The second input is an action-history vector ($h_{t}$) encoding the applied filters (actions) and their parameter values; this vector is processed by the LN layer. For the detailed architecture of the policy network, see Figure 1.

### 3.2. RL Environment Setup

The environment setup for RL is crucial for training the ARSIE agent to select better filters. In this sub-section, we describe the details of the RL environment.

**Figure 2:**
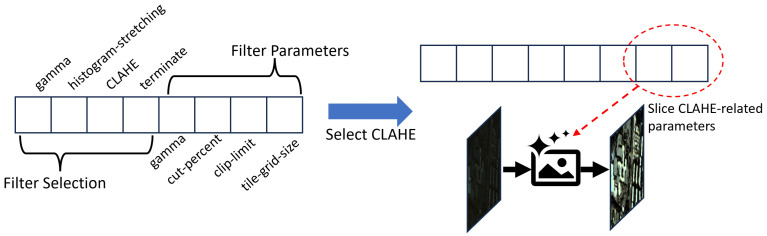
This figure illustrates the structure of the action vector: a filter–selection subvector concatenated with a filter–parameter subvector. The selection part is a probability vector over filters, while the parameter part encodes the per-filter settings. For example, for CLAHE we use two parameters—clip limit and tile grid size. In the example shown, when CLAHE is selected, the last two entries of the action vector are sliced to obtain the CLAHE parameters.

- state, $S_{t}$: Simply saying an image such as a degraded image or filtered image. Every first state in an episode is a degraded image and filtered images are followed after that. It is an input of the backbone network in policy.
- action, $a_{t}$: It consists of two parts: the discrete action vector and its parameter vector (continuous). All filter parameters are represented in the action vector at once. In the action phase, we select an action in discrete action vector $a_{t,f}$ and slice its parameter from parameter vector $a_{t,p}$: $a_{t}=\left[a_{t,f};a_{t,p}\right]=\pi \left(s_{t},h_{t}\right)$ (please see Figure 2). Detailed filter information is shown in the Section 4.
- action-history, $h_{t}$: The action-history mechanism prevents re-selecting the same action multiple times. $h_{t}$ represents all actions (filter information) to construct $s_{t}$, current state (image): $h_{t}=a_{1}+a_{2}+...a_{t-1}$. You can check that $h_{t}$ stores not only which actions are used but also the parameters of used filters. It serves as input to the policy network and plays a role as an action mask to suppress the repeated selection of filters. Please see Figure 2 to check the detailed structure.
- reward, $r_{t}$: The reward signal comes from the change in mIoU ($\Delta_{t}$) between consecutive states: we compute $\mathrm{mIoU}(S_{t})$ between the segmentation outputs of the current image ($S_{t}$) and the ground truth and $\mathrm{mIoU}(S_{t+1})$ between the filtered image ($S_{t+1}$) and ground truth.
  (1) $$\begin{array}{c} \Delta_{t}=\mathrm{mIoU}\left(S_{t+1}\right)-\mathrm{mIoU}\left(S_{t}\right). \end{array}$$
  The detailed description of reward follows after the list; we have proposed two types of reward design.
- state transition, $T(S_{t+1}|S_{t},a_{t},h_{t})$: The transition function updates the filtered image as the new current state.
- termination, $d_{t}\in \left\{0,1\right\}$: The agents do only terminate and the environment returns “done-signal” if all filters are used or agent reaches the state when $t\geq N_{avail}-1$; *t* is the processing timestamp and $N_{avail}$ is the maximum number of filters (actions) to process the image, $N_{avail}\leq N$; last action must be termination.

We design the reward function based on the difference mIoU. Basically, we find sequences of filters to improve the performance of the segmentation model. Therefore, the gains of mIoU are thought as the metric to measure the performance improvement of the segmentation model intuitively. We have two designs of reward. The first reward design is:

(2) $$r_{t}=R_{1}\left(\Delta_{t}\right)=\left\{\begin{array}{cc} w_{\mathrm{pos}}\;\Delta_{t}, & \mathrm{if}\;\Delta_{t}\geq \epsilon ,\\ w_{\mathrm{neg}}\;\Delta_{t}, & \mathrm{if}\;\Delta_{t}<\epsilon . \end{array}\right.$$

where $R_{1}$ means reward type 1, $w_{\mathrm{pos}}$ and $w_{\mathrm{neg}}$ are constant values to amplify the reward signal and ϵ is a threshold value. The second reward design is:

(3) $$\begin{array}{c} r_{t}=R_{2}\left(\Delta_{t}\right)=w_{\Delta }\Delta_{t}+w_{rel}\left(\mathrm{mIoU}\left(S_{t+1}\right)-\bar{\mathrm{mIoU}}\left(S_{t+1}\right)\right) \end{array}$$

where $R_{2}$ means reward type 2, $w_{\Delta }$ and $w_{rel}$ are constant values to amplify the reward signal (e.g., use 20,0.5 in experiments respectively) and $\bar{\mathrm{mIoU}}\left(S_{t+1}\right)$ is the average of batch states at step t+1. To generate batch states, we process the batch of degraded images at the episode start time.

The designs of $R_{1}$ and $R_{2}$ are intended to amplify the reward signal, because the actual mIoU lies in [0,1], whereas step-to-step changes in mIoU are typically much smaller, often within [0,0.1].

In $R_{1}$, we require the agent to achieve at least a minimal improvement by setting an ϵ-threshold. The intended effect of $R_{2}$ is used to compensate for variance due to image difficulty, so the agent can get meaningful rewards from the images which is hard to enhance.

To prevent reward over-expose, we clip the reward to [−1,1]. The experiment results from these two reward types are shown in the Section 4.

We train the ARSIE agent with Proximal Policy Optimization (PPO) [27]. PPO is a stable RL algorithm which incrementally trains the actor and critic of the agent; therefore, it has still been widely used until recently. The overall training procedure is described in Algorithm 1.
**Algorithm 1** Training loop for ARSIE**Input:** 
I, Y (segmentation ground truth), ϕ, $\phi_{bb}$, *F* (Available Filters), $M_{tr}$ (maximum iterations for training), $N_{avail}$**Output:** 
π (policy)  1:t←0  2:$S_{t}\leftarrow I$  3:$h_{t}\leftarrow 0$  4:Freeze weights of ϕ  5:**for** i←0 **to** $M_{tr}$ **do**  6:      (I,Y)← Get Data  7:      $\mathrm{mIoU}\left(S_{t}\right)\leftarrow$  CalculateMIoU (I,Y)  8:      **for** t←1 **to** $N_{avail}$ **do**  9:             $\left(C_{2},C_{3},C_{5}\right)\leftarrow$  GetFeatureMaps $\left(\phi_{bb}\left(S_{t}\right)\right)$10:           $\left(\mu_{2},\mu_{3},\mu_{5},\sigma_{5}\right)\leftarrow$  ComputeStats $\left(C_{2},C_{3},C_{5}\right)$11:           $x_{t}\leftarrow$  ConcatStats $\left(\mu_{2},\mu_{3},\mu_{5},\sigma_{5}\right)$12:           $a_{t}\leftarrow \pi \left(x_{t},h_{t}\right)$13:           $\left(f,f_{\mathrm{params}}\right)\leftarrow$  SelectFilter $\left(a_{t},F\right)$14:           $s_{t+1}\leftarrow$  ProcessImage $\left(S_{t},f,f_{\mathrm{params}}\right)$15:           $r_{t}\leftarrow$  GetReward $\left(\mathrm{mIoU}\left(S_{t}\right),\mathrm{mIoU}\left(S_{t+1}\right)\right)$16:                                  ▹ Fix the reward type17:           $h_{t}\leftarrow$  UpdateActionHistory $\left(a_{t}\right)$18:           $d_{t}\leftarrow$  CheckTermination (t,f)19:           Store data($S_{t},a_{t},S_{t+1},h_{t},r_{t},d_{t}$) to buffer20:           **if** $d_{t}=1$ **then**21:               Break the loop22:      Update policy π using collected trajectories23:                            ▹ Update policy using PPO24:**return** π

## 4. Experimental Results

### 4.1. Datasets

#### 4.1.1. K3A-CITY

K3A is the name of the Korean satellite—KOMPSAT-3A: Korea Multi-Purpose Satellite-3A. **K3A-CITY** consists of the satellite images for various locations in Korea (Sejong City and its surrounding areas) with labels: *background, building, road, plastic house, farmland, forest, waterside*. The satellite images have a 16-bit format, so we process the images using Histogram Stretching which clips 1% of top and bottom pixel values. The ground sampling distance (GSD) of **K3A-CITY** is 0.55 m. It consists of 8309 pieces of training data and 1269 pieces of testing data. This dataset is only used to train segmentation models.

#### 4.1.2. K3A-CITY-8bit

**Figure 3:**
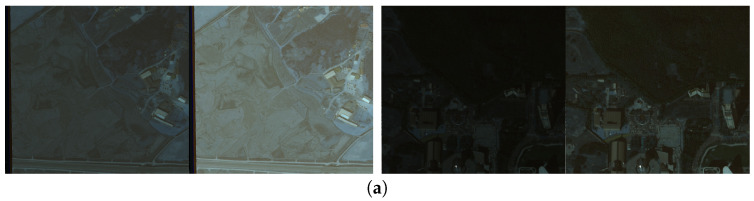

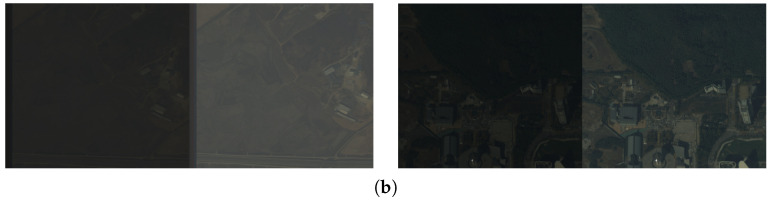
There are four pairs of examples for (**a**) K3A-CITY-8bit and (**b**) K3A-CITY-D. Detailed description of these datasets are in Section 4.1 For visibility, we display each original image together with a brightness-enhanced image.

**K3A-CITY-8 bit** is constructed by converting 16 bit K3A-CITY images to 8 bit by dividing pixel values by 256. This dataset seems to be like low-light filtered images. This is used only for conceptual proof of the proposed method (See Figure 3a). It consists of 3431 training data and 524 testing data.

#### 4.1.3. K3A-CITY-D

“D” in **K3A-CITY-D** stands for “Degradation”. For each image, we randomly apply a composition of one to three degradation types selected from *gain gamma, vignetting, haze, Poisson–Gaussian noise, motion blur, and Gaussian blur*. After applying degradations, we convert the images from 16-bit to 8-bit. Therefore, **K3A-CITY-D** has variously degraded images (See Figure 3b). This dataset contains the same number of data as **K3A-CITY**.

### 4.2. Segmentation Models

We train two segmentation models; **DeepLabV3+** [28] with **ResNet-101** [21] backbone and **PSP-Net** [29] with **U-Net** [30] backbone. As mentioned before, we utilize **K3A-CITY-8bit** dataset to train these models. In ARSIE agent training phase, the segmentation models are not updated; their model parameters are fixed.

### 4.3. Reinforcement Learning Settings

As mentioned before, we introduce two reward types. In Equation (3), we set $w_{\Delta }$ and $w_{rel}$ as 20.0 and 0.5 respectively. In Equation (2), we set $w_{pos}$ and $w_{neg}$ as 20.0 and 5.0 respectively. And ϵ in Equation (2) is set as 0.001. We find these constant values experimentally while we train the ARSIE model on **K3A-CITY-D**. We encourage the agent to find the sequence of filters to improve the segmentation performance, even if the performance gains are small. To do this, $w_{\Delta }$ and $w_{pos}$ become relatively higher values compared to $w_{rel}$ and $w_{neg}$. In view of traditional RL setting, multiple state processing is not allowed unless asynchronous RL setting [31,32]. However, we compute mIoU of image batch in parallel manner to reduce computation time cost. Except computing mIoU, we apply different filters to each image in the batch and transit each state in the batch one by one. We process multiple states (images) at once, but we need to notice that each of the states follows individual episodic trajectory; it is crucial to compute advantages and returns for Generalized Advantage Estimation (GAE). These trajectories are managed individually and inserted to the replay buffer. We use the number of images to be processed as 4, training batch size as 32 and size of episode replay buffer as 128.

### 4.4. Filters

We use a total of nine filters including terminate. Below is the description of the filter details with their parameter ranges:

- gamma [33]: Nonlinear brightness adjustment; γ<1 brightens shadows, γ>1 compresses highlights. γ∈[0.5,2.5]
- hist_stretch [34]: Global contrast expansion (percentile-based min–max); simple and fast. cut_percent ∈[0.0,3.0]
- unsharp_mask [35]: Edge sharpening via subtracting a blurred copy. kernel_size ∈{3,4,5,...,11},σ∈[0.5,3.0],amount∈[0.5,2.0]
- clahe [6]: Local contrast enhancement with a clip limit; robust to non-uniform illumination. clip_limit ∈[1.0,10.0], tile_grid_size ∈[4.0,16.0]
- bbhe [36]: Bi-histogram equalization preserving mean brightness; less color shift than standard HE.
- dsihe [37]: Median-based bi-histogram equalization; balances dark and bright regions.
- rmshe [38]: Recursive mean-separate equalization; improves fine tones but can over-amplify noise. recursion_depth ∈[1.0,4.0]
- wthe [39]: Weighted/thresholded histogram equalization; boosts contrast while limiting over-enhancement. α∈[0.1,2.0]
- terminate: No operation; stop further processing to avoid over-adjustment.

### 4.5. Analysis

We demonstrate the image enhancement performance of ARSIE in this section. However, we cannot compare the previous methods [18,19,20] with some reasons. Previous methods are mainly applicable to settings where the original images are available (e.g., computing PSNR during training or comparing against filtered outputs), or where evaluation is limited to object detection. In contrast, our method aims to produce enhanced images tailored to semantic segmentation, and it does so without access to original images during training. So we demonstrate our method compared to conventional image enhancement methods.

#### 4.5.1. Results on K3A-CITY-8bit and K3A-CITY-D

We report the experimental results of ARSIE on two datasets: **K3A-CITY-8bit** and **K3A-CITY-D**. Table 1 summarizes the performance of **DeepLabV3+**. On **K3A-CITY-8bit**, ARSIE consistently improves mIoU over the baseline.

#### 4.5.2. Action Sequence Characteristics

We further analyze the action sequences selected by the trained agents. For **K3A-CITY-8bit**, the learned policies tend to converge to a small set of sequences. In particular, agents trained with $R_{1}$ and $R_{2}$ frequently choose {clahe,gamma,terminate} and {clahe,hist_stretch,terminate}, respectively. This behavior is expected because **K3A-CITY-8bit** is generated only by bit-depth conversion, which resembles a single-type degradation; therefore, a nearly fixed enhancement recipe becomes effective. To mitigate the above limitation, we train ARSIE agents on **K3A-CITY-D**, which contains images with diverse degradation types and levels. On **K3A-CITY-D**, the agents exhibit substantially more diverse enhancement behaviors. For example, the agents with ($N_{avail}=5$, $R_{1}$) and ($N_{avail}=5$, $R_{2}$) select 77 and 24 unique action sequences, respectively, across 524 test images.

#### 4.5.3. Effect of $N_{avail}$

To study the impact of the action budget, we additionally train agents with ($N_{avail}=7$, $R_{2}$) and ($N_{avail}=9$, $R_{2}$) on **K3A-CITY-D**. We observe that these agents apply between at least 3 actions and at most $N_{avail}$ actions per image, indicating that the policies learn to avoid unnecessary operations by terminating early. However, both ($N_{avail}=7$, $R_{2}$) and ($N_{avail}=9$, $R_{2}$) yield lower mIoU than ($N_{avail}=5$, $R_{2}$). This suggests that training with a stricter constraint (i.e., a smaller, carefully chosen $N_{avail}$) can be more beneficial for constructing effective ARSIE agents on degraded datasets.

#### 4.5.4. Comparison with Heuristic Pipelines

We construct two heuristic pipelines, rmshe→clahe→gamma, hist_stretch→clahe→gamma. The results for heuristic pipelines are shown in Table 1. We compare ARSIE and heuristic pipelines in only **K3A-CITY-D** because ARSIE constructs fixed sequences of filters on **K3A-CITY-8bit**. These pipelines make enhanced results but these results cannot exceed the performance of ARSIE variants. This demonstrates that ARSIE finds appropriate filter sequences suitable for degraded images.

#### 4.5.5. Results with U-Net-Based PSPNet

Table 2 reports the results of ARSIE agents with the **U-Net**-based **PSPNet** backbone. The overall trends are consistent with those of **DeepLabV3+** in Table 1: mIoU improves by approximately 20.0, and $P_{EI}$ remains on a similar scale. For fair comparison, we use the same feature-map selection order as in the **DeepLabV3+** experiments during training.

#### 4.5.6. Segmentation Results for Filtered Images

**Figure 4:**
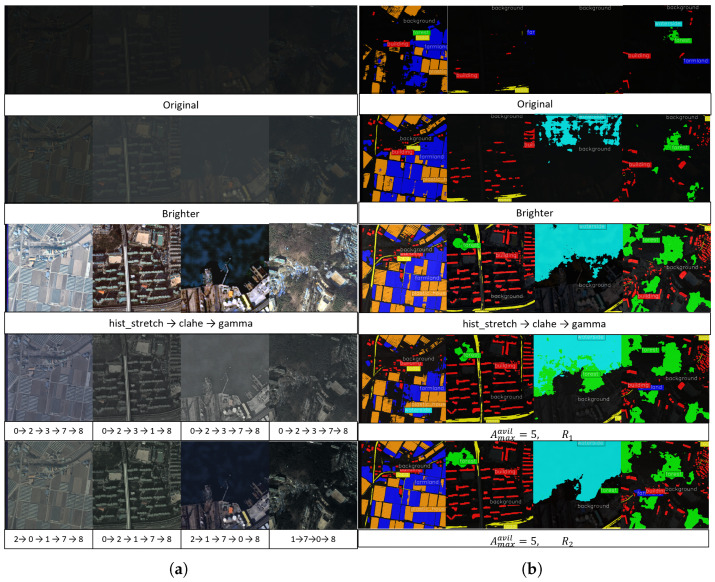
This figure shows image filtering results (**a**) generated by ARSIE agents with ($N_{avail}=5$, $R_{1}$) and ($N_{avail}=5$, $R_{2}$) and segmentation results (**b**) for (**a**). In sequence, each row contains four images: the original, a brightness-enhanced version (Brighter), heuristic pipeline (hist_stretch → clahe → gamma), the results from the agent with ($N_{avail}=5$, $R_{1}$), and the results from the agent with ($N_{avail}=5$, $R_{2}$). In rows 4 and 5, the numbers denote the filter (action) indices defined in Section 4.3: 0 = gamma, 1 = hist_stretch, …, 8 = terminate. Also, the left one is an earlier used filter and the filters are used sequentially. **Label colors:** background (black), building (red), road (yellow), water (sky blue), forest (green), farmland (blue), and plastic house (orange).

Figure 4 shows enhanced images processed by the filters selected from ARSIE agents with ($N_{avail}=5$, $R_{1}$) and ($N_{avail}=5$, $R_{2}$) and their segmentation results. In row 3 in Figure 4a, the agent with ($N_{avail}=5$, $R_{1}$) selects almost 5 filters (including terminate); the images have low contrast. However, the agent with ($N_{avail}=5$, $R_{2}$) has a case of using 4 filters in row 4; also the images are clearer and more colorful than the images in row 3. Since $R_{2}$ directly gives the negative rewards, we can conclude that the agent avoids selecting negative-effected filters during processing and unnecessary filters. The segmentation results (Figure 4b) for the processed images show the performance gains; the segmentation model can find a more desired segmentation area from the filtered images than the predictions from the only brightness-enhanced images. When comparing the 3rd prediction results in row 3 and 4, the result from row 4 has fewer false-positive pixels than the one from row 3. The numerical denoising results of image enhancement are described in Appendix B.

#### 4.5.7. Computational Costs

We measured the runtime of a single classical filter and ARSIE on the same device (Intel(R) Core(TM) i9-10900X CPU @ 3.70 GHz, NVIDIA RTX 4090, and 256 GB RAM). On average, ARSIE required 8.7–9.1 s per image when executing four filters followed by terminate (i.e., five actions in total). Executing a single classical filter required 1.7–1.9 s, resulting in approximately 6.8 s for four filters. Therefore, the additional overhead introduced by ARSIE (policy inference and control logic) was about 1.6 s per image, with a average GPU memory usage of approximately 2.4 GB. To reduce inference time for the segmentation model, we use AMP (Automatic Mixed Precision package) with **BFfloat16**.

## 5. Conclusions

We have studied the automatic and adaptive enhancement of remote-sensing images acquired under adverse conditions with the goal of improving semantic segmentation performance. To this end, we have proposed ARSIE (Adaptive Remote Sensing Image Enhancement), which automatically selects a sequence of classical filter operations for degraded images. We have demonstrated that ARSIE agents choose image-specific filter sequences, producing enhanced images that improve segmentation predictions and remain visually interpretable (see Figure 4).

Despite these encouraging results, several limitations remain and motivate future work. First, the current filter pool primarily consists of brightness- and contrast-related operators. A more comprehensive pool grounded in the physical characteristics of remote-sensing imagery (e.g., atmospheric effects and sensor-specific artifacts) should be constructed, and the agent should be trained to select only necessary operations from this pool. Second, we have found that constraining the action budget via $N_{avail}$ is important: without this constraint, performance gains have been modest and the agent has tended to apply redundant filters. Although the constraint mitigates over-processing and helps prevent overfitting, a more principled mechanism for adaptive early termination and sequence-length control is required. Third, the proposed framework requires a segmentation model during both training and inference, which increases computational cost and runtime. Reducing this overhead is essential for practical deployment, including potential on-board computing scenarios. Finally, $P_{EI}$ does not reach 100%, indicating that enhancement is not consistently beneficial across all inputs. We hypothesize that some samples exhibit severe or atypical degradations that are not sufficiently addressed by the current degradation modeling and filter pool. Future work will investigate improved degradation strategies and will evaluate the method on real-world noisy imagery to better understand failure cases and improve robustness.

## Figures and Tables

**Table 1:** This table shows the performance of ARSIE while using **DeepLabV3+**. We describe the ARSIE model with reward type. $P_{EI}$ means the proportion of mIoU-enhanced images in the test set. clahe model means the result when the images are applied only once by clahe.

Dataset	Model	$N_{\mathrm{avail}}$	mIoU	$P_{\mathrm{EI}}$
K3A-CITY-8bit	Baseline (no filter)	-	34.76	-
	clahe	-	38.39	48.85%
	ARSIE ($R_{1}$)	3	45.46	**83.78**%
	ARSIE ($R_{2}$)	3	**46.26**	82.06%
K3A-CITY-D	Baseline (no filter)	-	15.69	-
	clahe	-	31.93	86.64%
	rmshe → clahe → gamma	-	25.68	80.92%
	hist_stretch → clahe → gamma	-	36.42	77.29%
	ARSIE ($R_{1}$)	5	38.81	87.59%
	ARSIE ($R_{2}$)	5	**40.05**	**92.94%**
	ARSIE ($R_{2}$)	7	38.66	90.65%
	ARSIE ($R_{2}$)	9	35.54	89.69%

**Table 2:** This table shows the performance of ARSIE while using **PSPNet**. We describe the ARSIE model with reward type. $P_{EI}$ means the proportion of quality-enhanced images in the test set.

Dataset	Model	*N_avail_*	mIoU	P*_EI_*
K3A-CITY-D	Baseline (no filter)	-	14.31	-
	ARSIE ($R_{1}$)	5	34.94	92.37%
	ARSIE ($R_{2}$)	5	35.82	93.13%

**Figure A1:**
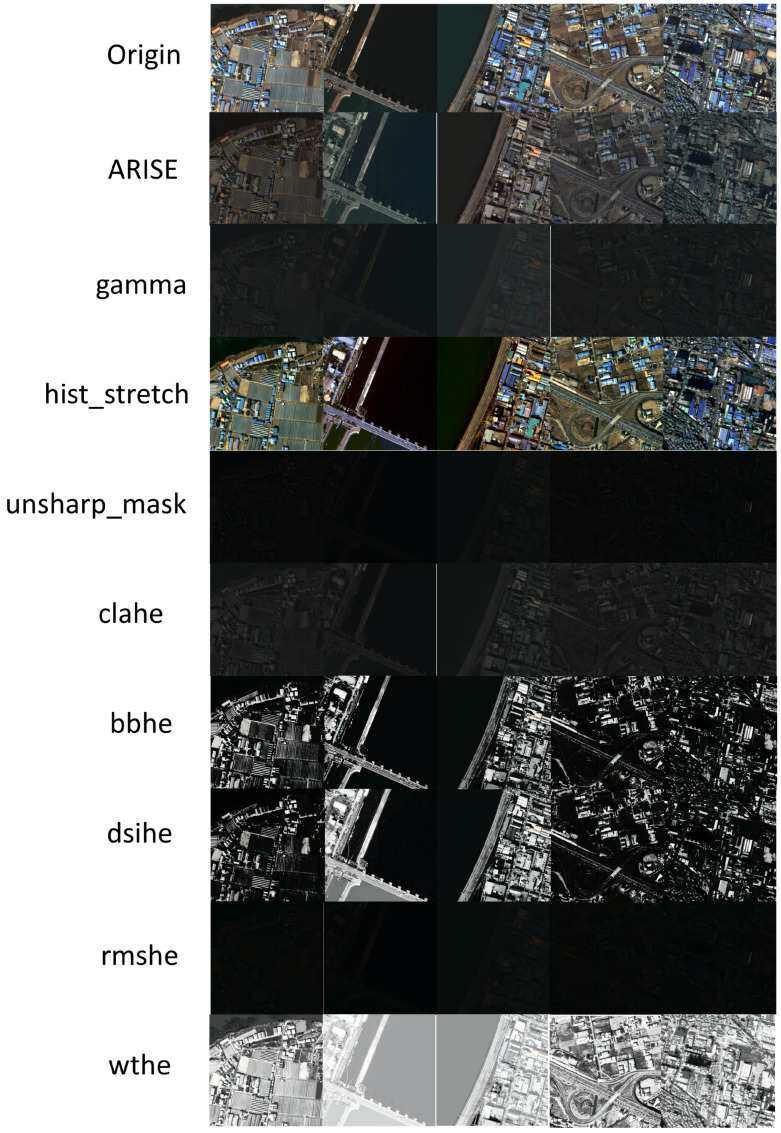
Enhancement output of ARSIE and all single filter models. “Origin” images are described in Appendix B and other images are processed from **K3A-CITY-D**. We chose five different images and model (filter) names are described on the left-hand side.

## Data Availability

The data presented in this study are available from the corresponding author on reasonable request, as they are not publicly distributable due to restrictions from the data provider (KARI; Korea Aerospace Research Institute, the government research institution).

## Appendix A. Comparison to Single Filter Models

We showed the numerical results from single filter models. We made single filter models which apply the image to a filter described in Section 4.4 only once.

We used the filter parameter values as the median value of each filter parameter range described in Section 4.4. In Table A1, all single filter models did not always improve the images well. In **K3A-CITY-8bit**, some models had worse average performance than Baseline. It means that some filters are not proper for **K3A-CITY-8bit**. $P_{EI}$ values was limited about 57%; best $P_{EI}$ was 56.30% in unsharp_mask. In **K3A-CITY-D**, there was no loss of average performance. Some models achieved $P_{EI}$ above 70%. However, all cases could not exceed the performance of ARSIE model results.

**Table A1:** This table shows the performance of a single filter model while using **DeepLabV3+**. $P_{EI}$ means the proportion of quality-enhanced images in the test set.

Dataset	Model	*N_avail_*	mIoU	*P_EI_*
K3A-CITY-8bit	ARSIE ($R_{2}$)	3	**46.26**	82.06%
	gamma	-	30.97	20.61%
	hist_stretch	-	35.75	48.28%
	unsharp_mask	-	39.24	56.30%
	clahe	-	38.39	48.85%
	bbhe	-	31.54	28.82%
	dsihe	-	31.09	27.67%
	rmshe	-	36.53	40.65%
	wthe	-	25.64	22.90%
K3A-CITY-D	ARSIE ($R_{2}$)	5	**40.05**	**92.94%**
	gamma	-	22.13	74.43%
	hist_stretch	-	39.31	80.53%
	unsharp_mask	-	20.16	80.92%
	clahe	-	31.93	86.64%
	bbhe	-	21.07	67.18%
	dsihe	-	22.87	68.89%
	rmshe	-	18.01	52.67%
	wthe	-	20.49	60.30%

## Appendix B. The Numerical Image Enhancement Results

We estimate how much an image is enhanced by using the Structure Similarity Index Measure (SSIM) and Peak Signal-to-Noise Ratio (PSNR) [40]. These two metrics are used to evaluate the similarity between the processed image and the original image. However, we construct **K3A-CITY-D** from original remote sensing images which are 16-bit. This means that we cannot directly compare the original images and the processed images in the manners of SSIM and PSNR. Therefore, we converted the 16-bit original images to the 8-bit ones with 1% histogram stretching because this processing is actually used to train the model. Simply saying, we measure the similarity between training data for segmentation model and the processed images.

Table A2 shows the results of PSNR and SSIM for ARSIE and single filter models. We find that segmentation performance improvement is not proportional to PSNR and SSIM. As mentioned before, ARSIE is more suitable to improve segmentation performance than the others. However, ARSIE does not have the best PSNR and SSIM. Also, rmshe is the worst model in the manner of segmentation performance, but it has higher values of PSNR and SSIM. This situation seems to be came from not considering PSNR and SSIM metrics in the training phase. In view of segmentation model, PSNR and SSIM are not always suitable to measure the image enhancement because the easier images for segmentation cannot be similar to the images suitable and interpretable for humans. Figure A1 shows the visual results for all models including original images. hist_stretch which has higher PSNR and SSIM is very similar to original images but the images from ARSIE are a little different from original images.

**Table A2:** This table shows PSNR and SSIM for ARSIE and single filter models.

Dataset	Model	*N_avail_*	PSNR	SSIM
K3A-CITY-D	ARSIE ($R_{2}$)	5	12.63	0.15
	gamma	-	14.17	0.27
	hist_stretch	-	10.02	0.08
	unsharp_mask	-	13.03	0.19
	clahe	-	14.08	0.25
	bbhe	-	9.64	0.10
	dsihe	-	9.78	0.10
	rmshe	-	12.93	0.18
	wthe	-	5.61	0.06
